# DENV-1 Titer Impacts Viral Blocking in *w*Mel *Aedes aegypti* with Brazilian Genetic Background

**DOI:** 10.3390/v16020214

**Published:** 2024-01-31

**Authors:** Jessica Corrêa-Antônio, Mariana R. David, Dinair Couto-Lima, Gabriela Azambuja Garcia, Milan S. G. Keirsebelik, Rafael Maciel-de-Freitas, Márcio Galvão Pavan

**Affiliations:** 1 Laboratório de Mosquitos Transmissores de Hematozoários, Instituto Oswaldo Cruz, Fiocruz, Rio de Janeiro 21040-900, Brazil; jessicaacorrea@gmail.com (J.C.-A.); maridavid@ioc.fiocruz.br (M.R.D.); dcouto@ioc.fiocruz.br (D.C.-L.); gabiazambuja@hotmail.com (G.A.G.); milankeirsebelik96@gmail.com (M.S.G.K.); freitas@ioc.fiocruz.br (R.M.-d.-F.); 2 Department of Arbovirology, Bernhard Nocht Institute of Tropical Medicine, 20359 Hamburg, Germany

**Keywords:** *Ae. aegypti*, *Wolbachia*, *w*Mel, DENV-1, vector competence, dengue

## Abstract

Several countries have been using *Wolbachia* deployments to replace highly competent native *Aedes aegypti* populations with *Wolbachia*-carrying mosquitoes with lower susceptibility to arboviruses such as dengue, Zika, and chikungunya. In Rio de Janeiro, *Wolbachia* deployments started in 2015 and still present a moderate introgression with a modest reduction in dengue cases in humans (38%). Here, we evaluated the vector competence of wild-type and *w*Mel-infected *Ae. aegypti* with a Brazilian genetic background to investigate whether virus leakage could contribute to the observed outcomes in Brazil. We collected the specimens in three areas of Rio de Janeiro with distinct frequencies of mosquitoes with *w*Mel strain and two areas with wild *Ae. aegypti*. The mosquitoes were orally exposed to two titers of DENV-1 and the saliva of DENV-1-infected *Ae. aegypti* was microinjected into *w*Mel-free mosquitoes to check their infectivity. When infected with the high DENV-1 titer, the presence of *w*Mel did not avoid viral infection in mosquitoes’ bodies and saliva but DENV-1-infected *w*Mel mosquitoes produced lower viral loads than *w*Mel-free mosquitoes. On the other hand, *w*Mel mosquitoes infected with the low DENV-1 titer were less susceptible to virus infection than *w*Mel-free mosquitoes, although once infected, *w*Mel and *w*Mel-free mosquitoes exhibited similar viral loads in the body and the saliva. Our results showed viral leakage in 60% of the saliva of *w*Mel mosquitoes with Brazilian background; thus, sustained surveillance is imperative to monitor the presence of other circulating DENV-1 strains capable of overcoming the *Wolbachia* blocking phenotype, enabling timely implementation of action plans.

## 1. Introduction

Arthropod-borne viruses (arboviruses) such as dengue (DENV), Zika (ZIKV), and chikungunya (CHIKV) have spread in urbanized areas around the world, representing a significant public health burden, with 3.9 billion people at risk in 129 countries [1]. Dengue is responsible for more than 90% of the arboviral cases reported in the Americas, reaching 2.8 million cases in 2022 [2,3]. *Aedes aegypti* (Diptera: Culicidae) is the primary vector, and it is highly associated with anthropogenic environments, where female mosquitoes lay eggs mainly in artificial containers inside or near human habitations and feed preferentially on humans [4,5].

Due to a lack of a vaccine broadly available for many arboviruses, the most recommended way to mitigate transmission is through intensifying vector control. Traditional control methods, such as the search for and elimination of breeding sites and the use of insecticides, present limited effectiveness in avoiding outbreaks [6]. Given this challenging scenario, designing alternative effective interventions is critical to reducing the disease burden. One of these strategies is the release of *Ae. aegypti* infected with the intracellular symbiont *Wolbachia pipientis* (Rickettsiales, Anaplasmataceae). This bacterium is naturally present in nearly 40% of arthropods but not in *Ae. aegypti* [7]. Its use is based on at least three phenotypes to support introgression into native mosquito populations: reduction in arbovirus replication and transmission in mosquitoes [8,9,10], maternal transmission (MT), and capacity to induce cytoplasmic incompatibility (CI) [11]. CI results in embryonic mortality in crosses between *Wolbachia*-free females and *Wolbachia*-infected males but not between females with *Wolbachia* and males with or without the bacteria [12,13].

Among the *Wolbachia* strains currently introgressed into *Ae. aegypti*, *w*Mel (from *Drosophila melanogaster*) does not seem to influence mosquito fecundity and survival, but it reduces the oviposition success and the viability of eggs stored for many weeks under laboratory conditions [14,15,16]. Regarding the refractoriness to arboviruses, the *w*Mel strain inhibits the transmission of CHIKV [8], Yellow Fever [17], and Mayaro viruses [18], but its protective effect is not complete against DENV-1 [19,20,21] and ZIKV [22]. Although the complete mechanism of *Wolbachia*-mediated blocking of arboviruses is not fully understood, there is evidence of mosquito immune priming, competition for lipids, and production of reactive oxygen species, among others (reviewed in [23,24]). The inhibition of arbovirus replication seems to also be bacterial density dependent, i.e., higher blocking is expected in individuals with higher *Wolbachia* density in mosquito tissues [25].

*Wolbachia* deployments in *Ae. aegypti* populations have been achieving promising outcomes around the globe. A randomized control trial in Yogyakarta, Indonesia, showed >70% *w*Mel invasion after 4–7 months of release and a 77% reduction in dengue cases [26]. In North Queensland, Australia, dengue incidence reduced nearly 65% during *w*Mel releases for 28 months between 2014 and 2017 and >95% 24 months after releases [27]. Another recent study in Colombia has shown that the *Wolbachia* deployments occurring since 2015 achieved an impressive dengue incidence decrease of 94–97%, with two out of the three cities that received the program being considered fully treated (60% of the local mosquitoes with *Wolbachia* [28,29]).

The first release of the *w*Mel strain in Rio de Janeiro was conducted in 2015 at Tubiacanga, an isolated locality with around 750 premises. The release consisted of approximately 10,000 females/week for a period of 24 weeks. The *w*Mel frequency in the field reached 80% in the 18th week of releases, and it remained between 85 and 95% one year after ceasing the releases [14]. From 2017 to 2019, the release areas were expanded, covering a total area of 86.8 km^2^ with around 890,000 inhabitants [30]. Niterói (13 km away from Rio de Janeiro) was the first city in Brazil to adopt citywide deployment of *Wolbachia* [31]. After a 2-year release program, the frequency of *w*Mel-infected *Ae. aegypti* across the city varied from 33 to 90%, and an overall reduction of 69% in dengue cases was reported [32]. A more modest outcome was obtained in the city of Rio de Janeiro. After releasing 67 million *w*Mel *Ae. aegypti* mosquitoes between 2017 and 2019, a 32% *w*Mel introgression into the wild population was achieved, and a reduction of 38% and 10% of dengue and chikungunya notifications were reported, respectively [30]. The results of Rio de Janeiro are of interest, but a more in-depth investigation is warranted to assess the obstacles that limited *w*Mel introgression and the reduction in arbovirus cases.

Several laboratory studies have been evaluating the effect of *Wolbachia* on the vector competence of *Ae. aegypti* with different genetic backgrounds to DENV strains, but most of them challenged mosquitoes with high doses of the virus (i.e., unrealistic viral loads) to ensure mosquito infection [19,20,21,33,34,35,36]. For instance, *w*Mel *Ae. aegypti* mosquitoes with a Brazilian genetic background showed refractoriness to high doses of DENV-1 (10^6^ and 10^7^) and ZIKV (10^6^ and 10^8^) viruses [32]. In the field, *Ae. aegypti*, however, can also transmit arboviruses when fed the blood of infected vertebrates with low viremia [37]. Moreover, different genetic backgrounds of arboviruses and mosquitoes circulate in the field, which ultimately can result in contrasting infection and transmission scenarios. Herein, we provide an independent dataset involving vector competence assays to investigate the capacity of *w*Mel, *w*Mel-free, and wild *Ae. aegypti* collected in five locations of Rio de Janeiro with different invasion patterns to become infected and transmit DENV-1 through experimental oral infections using low and high viral titers.

## 2. Materials and Methods

### 2.1. Study Areas

Rio de Janeiro’s capital comprises 1200 km^2^, and it is spatially heterogeneous, with 163 neighborhoods where approximately 6.2 million people live [38]. *Wolbachia* releases first started in 2015 and ceased in 2021, covering 29 neighborhoods that represent 17.8% of the city [39]. Niterói is a city connected to Rio de Janeiro by a bridge across Guanabara Bay and has an area of 133 km^2^, with 482 thousand inhabitants and 52 neighborhoods [40]. *w*Mel releases first started in Niterói in late 2015, and in 2021 75% of the territory has already received *Wolbachia* [41].

**Figure 1:**
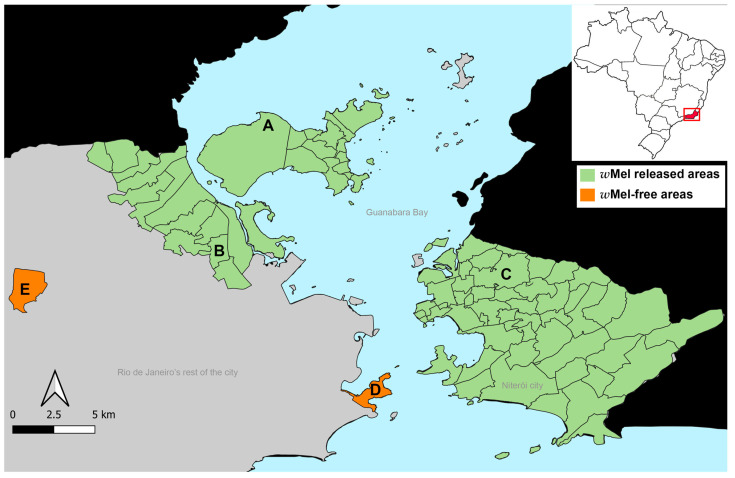
Study areas in Rio de Janeiro. A—FI (Tubiacanga); B—PI1 (Bonsucesso); C—PI2 (Fonseca); D—NR1 (Urca); E—NR2 (Vila Valqueire). In green, *w*Mel current released areas, and in orange, *w*Mel-free areas.

*Ae. aegypti* mosquitoes were sampled in five areas of Rio de Janeiro from September to early November 2019 (Figure 1), three of which with a previous history of *Wolbachia* releases: (A) Tubiacanga, Rio de Janeiro (22°47′06″ S; 43°13′32″ W), received *w*Mel in 2015 and since then the *w*Mel frequency has been near 100% (hereafter “FI” (Full Invasion)) [42]; (B) Bonsucesso, Rio de Janeiro (22°51′44″ S; 43°15′14″ W), received *w*Mel in early 2019, ~6 months before mosquito samplings and in late 2019 the *w*Mel frequency was ~40% (“PI1” (Partial Invasion)) [8], and (C) Fonseca, Niterói (22°52′37″ S; 43°4′32″ W), where *w*Mel deployments occurred in late 2019, the same period as mosquito samplings (“PI2”), when *w*Mel frequency was ~60% [31]. We also obtained mosquitoes from Urca (22°57′15″ S; 43°10′3″ W; D), a neighborhood of Rio de Janeiro distant from the *w*Mel release areas, and from where mosquitoes were collected to be backcrossed with *w*Mel *Ae. aegypti* to produce mosquitoes with local genetic backgrounds; therefore, wild *Ae. aegypti* (“NR1” (No *w*Mel Releases)) and Vila Valqueire, Rio de Janeiro (22°52′40″ S; 43°21′47″ W; E), where no *w*Mel strain releases occurred (i.e., wild *Ae. aegypti*) were not used in the backcrosses (hereafter “NR2”).

Two different sampling strategies were conducted simultaneously between September and November 2019. Using backpack aspirators, we captured 25 adult female *Ae. aegypti* in each of the study areas to check the prevalence and relative density of *w*Mel in field mosquitoes. At the same time, 60 ovitraps were evenly distributed to obtain a minimum of 5000 eggs per area that best represents the genetic diversity of *Ae. aegypti* in the field. Five hundred eggs were hatched in plastic containers (45.5 cm × 28 cm × 7.7 cm) according to their area with 3 L of water and yeast, and larvae were fed daily with 4.5 mg of TetraMin^®^ fish food (Tetra, Melle, Germany) until the pupae stage. Adult mosquitoes were identified using taxonomic keys [43]. Those identified as *Ae. aegypti* formed site-specific lab colonies that were kept under insectary conditions (80 ± 5% humidity and 25 ± 3 °C) with sugar solution (10%) *ad libitum* until 24 h before DENV infection.

### 2.2. Wolbachia DNA Detection and Quantification in Ae. aegypti

The DNA of *Wolbachia* was extracted as previously described [10] from both field-caught adults and lab-reared *Ae. aegypti* mosquitoes. *w*Mel infection status was determined through a duplex PCR assay, which amplifies the *Ae. aegypti* ribosomal protein S17 (*RpS17*) as an internal mosquito control, and also the *Wolbachia WD0513* gene to detect the *w*Mel strain [19]. Relative quantification was performed with TaqMan^TM^ Fast 1-Step Master Mix (Applied Biosystems, Waltham, MA, USA), using the QuantStudio^TM^ 6 Flex Real-Time PCR System (Applied Biosystems, Waltham, MA, USA) as previously published [44]. Five individuals from NR1 and NR2 were used as negative controls.

### 2.3. Viral Strain and Oral Infections

Dengue-1 MV17 strain, isolated from a human case in 2015 at Minas Gerais (DENV1/*Homo sapiens*/Brasil/Contagem/MG/MV17/2015) [45], was obtained after four passages in C6/36 cell culture. The virus was inoculated in C6/36 cell culture 5 days before the experimental infections. F1 adult *Ae. aegypti* were kept in cages with access to sugar solution (10%) until they were 6–7 days old. They were deprived of sugar solution 24 h before blood feeding, which consisted of 1 mL of erythrocytes + 1 mL of fresh virus suspended in L15 medium for the infected group and 1 mL of erythrocytes + 1 mL of L15 medium for the uninfected groups. Female mosquitoes from the five populations were orally fed using an artificial feeder (Hemotek, Great Hardwood, UK) with human blood (approved by Fiocruz Ethics Committee—CAAE 53419815.9.0000.5248) at 37 °C for approximately 30 min. Only visually fully engorged females were selected for the analyses. Two viral titers of the same virus culture were offered: 6 × 10^8^ FFU/mL (focus-forming units/mL; hereafter called ‘high titer’) or 3 × 10^4^ FFU/mL (hereafter called ‘low titer’). The two viral titers were chosen as representative of natural viral loads from naturally infected human hosts [37]. While the females were feeding, a virus aliquot was serially diluted and inoculated in C6/36 cells. Viral envelope protein E was detected by immunofluorescence in C6/36 cultures using DENV-specific monoclonal antibodies (purified from ascitic fluid anti-dengue virus 1; in-house lab LATAM production; product batch: 041118FDEN1P; technical expert: Tiago Pereira). The focus-forming units (FFUs) were counted in an EVOS^®^ FL Auto Imaging System (Life Technologies, Carlsbad, CA, USA).

### 2.4. Mosquito Saliva Collection and Intrathoracic Microinjection

Mosquito females were anesthetized on ice, and their wings and legs were removed to collect their saliva at 14 dpi. Their proboscises were individually inserted into sterile filtered 10 µL pipette tips containing 10 µL of sterile Fetal Bovine Serum (Gibco, Thermo Fisher Scientific Inc., Waltham, MA, USA) mixed with blue food coloring and allowed to salivate for 30 min. Only the saliva of mosquitoes with blueish abdomens was collected. The F1 generation of *Ae. aegypti* females from NR1 were intrathoracically injected with 69 nL of saliva collected from DENV-1-infected females of FI, PI1, PI2, NR1, and NR2. Uninfected saliva from NR1 females was also inoculated as negative controls. Injections were carried out with a Nanoject II (Drummond Scientific Company, Broomall, PA, USA), as previously described [8]. 

Each saliva sample was inoculated in 15 female mosquitoes. Nineteen saliva samples of wild mosquitoes (NR1), 26 from areas with recent releases of *w*Mel (PI1 = 10, PI2 = 16), and 13 from an area with early *w*Mel releases (FI) were injected into 870 NR1 7-day-old female mosquitoes susceptible to DENV-1 [45]. We inoculated saliva only of those females that had their bodies positive for DENV-1 through RT-qPCR (excepting the negative controls). The injected female mosquitoes were killed seven days after saliva inoculations, and DENV-1 was screened through RT-qPCR (see next section for details). Saliva samples that produced at least one subsequent infection in the injected mosquitoes were classified as infective. In the case of females subjected to saliva microinjection, we assumed that susceptible mosquitoes would exhibit elevated viral loads in their bodies when exposed to saliva containing higher concentrations of infective virus. In essence, we postulated a positive correlation between the viral load in saliva-microinjected female bodies and the infective DENV load in the saliva.

### 2.5. DENV-1 RNA Detection and Quantification in Mosquitoes

The RNA of each sample was extracted individually from whole mosquitoes with a QlAamp Viral RNA Mini kit (Qiagen, Hilden, Germany) 14 dpi and seven days after intrathoracic injections. Detection and quantification of viral RNA was performed with TaqMan^TM^ Fast Virus 1-Step Master Mix (Applied Biosystems, Waltham, USA), using the QuantStudio^TM^ 6 Flex Real-Time PCR System (Applied Biosystems, Waltham, USA). Each reaction was made with previously published primers and probes [46]. Amplification conditions consisted of 12 nmoles of forward and reverse primers, 9 nmoles of probe, 5 µL of TaqMan^TM^ Fast Virus 1-Step Master Mix (Applied Biosystems), and 5 µL of RNA. Cycling conditions were as follows: 45 °C for 15 min, 95 °C for 20 s, followed by 40 amplification cycles of 95 °C for 15 s, 58 °C for 5 s, and 60 °C for 30 s. Viral copy numbers were calculated by interpolation onto an internal standard curve made up of a six-point dilution series (10^1^–10^6^ FFU/mL) of DENV-1.

### 2.6. Statistical Analysis

DENV-1 loads in whole mosquitoes (14 dpi) and in susceptible mosquitoes intrathoracically injected with mosquito saliva (7 dpi) were compared according to their collected area, *Wolbachia* presence and density, and to the two DENV-1 titers (3 × 10^4^ FFU/mL and 6 × 10^8^ FFU/mL). The distribution of DENV-1 load was not normally distributed (Shapiro–Wilk test = 0.85, *p* < 0.001) and, therefore, was compared through Wilcoxon–Mann–Whitney tests in the R environment [47]. Moreover, logistic and linear regressions were used to estimate the effects of *w*Mel and the viral titer on the infection status of mosquito bodies and saliva infectivity. The infection rate of mosquitoes was calculated as the number of mosquitoes infected with DENV-1 divided by the total number of mosquitoes tested. Linear regressions were also performed to infer the association between *Wolbachia* density and DENV-1 load. These analyses were performed with the ‘glm’ function using the binomial or Gaussian distributions in the R environment.

## 3. Results

### 3.1. Prevalence and Density of wMel in Field and Lab Ae. aegypti Mosquitoes

We accessed the prevalence of *Wolbachia* in the five areas, three where *w*Mel deployments took place before conducting the vector competence analyses (PI1, PI2, and FI; cf. Methods section for further details). The *w*Mel strain was detected in 41 out of 75 mosquitoes (an overall frequency of 54.7%) of the field populations where *w*Mel deployments occurred (Figure S1). All mosquitoes from FI (25/25) had the bacteria, while PI1 and PI2 had 20% (5/25) and 44% (11/25) of mosquitoes positive for *w*Mel, respectively. Pairwise comparisons did not show significant differences between *w*Mel densities in field-caught mosquitoes from the three deployed areas (W = 15; W = 107; W = 77 *p* > 0.05; Table S1). As expected, NR1 and NR2 mosquitoes were all *w*Mel-free. 

Considering the lab-reared F1 *Ae. aegypti* mosquitoes used in DENV-1 experimental infections, *w*Mel was detected in 137/185 samples (74%; Figure S1): 65/65 from FI (100%), 17/60 (28.3%) from PI1 and 55/60 (91.7%) from PI2. NR1 and NR2 mosquitoes were negative for *w*Mel. In general, lab-reared mosquitoes showed a higher relative density of *w*Mel when compared with field mosquitoes (W = 64; *p* < 0.001; Figure S1, Table S1).

### 3.2. wMel Detection in Ae. aegypti Mosquitoes Orally Exposed to Two DENV-1 Titers

**Figure 2:**
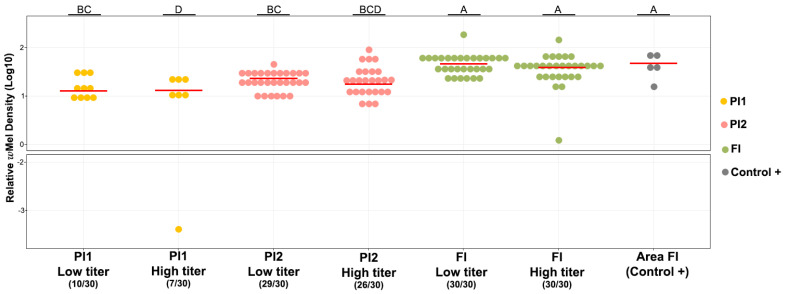
Relative density of *w*Mel in *Ae. aegypti* mosquitoes infected with DENV-1 at low (3 × 10^4^ FFU/mL) and high titers (6 × 10^8^ FFU/mL). The numbers inside parentheses indicate the number of *w*Mel-positive mosquitoes out of the total mosquitoes tested. Different letters (above the graph) indicate statistically significant differences in *w*Mel density (Wilcoxon–Mann–Whitney test; *p* < 0.05). Horizontal red bars represent the medians. Yellow circles represent samples infected with *w*Mel from PI1, pink circles from PI2, green circles from FI, and gray circles from *w*Mel-positive control (FI).

Considering the F1 mosquitoes used for oral infections with DENV-1, all *Ae. aegypti* from FI (where deployments started in 2015) were positive for *w*Mel (30/30; both viral titers; Figure 2). Likewise, specimens from PI2, where releases started in late 2019, had *Wolbachia* in 86.7% (26/30) and 96.7% (29/30) of mosquitoes when subjected to oral feeding with high and low DENV-1 titers. Despite the *Wolbachia* deployments taking place in PI1 before PI2 (early and late 2019, respectively), *Ae. aegypti* from PI1 subjected to DENV-1 high and low titer infections exhibited *w*Mel in only 23.3% (7/30) and 33.3% (10/30) of the total mosquitoes analyzed, respectively. 

Mosquitoes collected in FI presented higher relative *w*Mel density than mosquitoes from PI1 and PI2, where releases started five years later (W = 17–173.5, *p* < 0.001; Figure 2, Table S1). DENV-1-infected and non-infected mosquitoes had similar relative *w*Mel densities. Regarding only DENV-1-infected specimens, linear regression analyses evidenced no correlation between *w*Mel density and DENV-1 load (*F* = 2.354; *p* > 0.05 e R^2^ adjusted: 0.016, df = 80; Figure S2A). 

### 3.3. Vector Competence Assays

A total of 216 *Ae. aegypti* F1 from FI, PI1, and PI2 and 144 mosquitoes from NR1 and NR2 were exposed to DENV-1 oral infections with low and high titers, and their whole bodies were tested for infection via RT-qPCR. Ten *w*Mel and wild mosquitoes (from FI and NR1, respectively) non-exposed to DENV-1 were used as negative controls for arbovirus detection.

**Figure 3:**
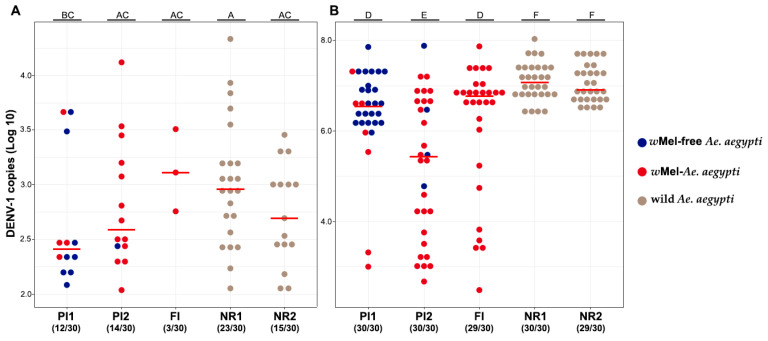
Number of DENV-1 copies in the whole body of *w*Mel, *w*Mel-free, and wild *Ae. aegypti* mosquitoes 14 days after the infection with the low titer (3 × 10^4^ FFU/mL) (**A**) and the high titer (6 × 10^8^ FFU/mL) (**B**). The numbers inside parentheses indicate the number of DENV-1-positive mosquitoes out of the total mosquitoes tested per area. Different letters (above the graph) indicate statistically significant differences (Wilcoxon–Mann–Whitney test; *p* < 0.05). Horizontal red bars represent the medians. Blue circles represent *w*Mel-free mosquitoes, red circles represent mosquitoes with *w*Mel, and brown circles represent wild mosquitoes. All negative controls for DENV-1 infection were negative.

A total of 30 individuals from each locality were analyzed for each virus titer. In general, whole-body *w*Mel mosquitoes had a lower DENV-1 infection rate than *w*Mel-free mosquitoes (GLM: *Wolbachia* (Yes) estimate = −1.2, *p* = 0.0005; Figure 3A, Table S2). Regarding the low titer, PI1 had 12 positives for DENV-1 (40%), of which 4/10 were *w*Mel *Ae. aegypti* and 8/20 were *w*Mel-free mosquitoes. PI2 had 14 mosquitoes infected with DENV-1 (45.2%), of which 13/29 were *w*Mel *Ae. aegypti* and 1/1 was a *w*Mel-free mosquito. The FI had only 3/30 *w*Mel mosquitoes infected with DENV-1 (10%). NR1 had 23/30 (76.7%) wild *Ae. aegypti* DENV-1 infected, while NR2 had 15/30 (50%) wild *Ae. aegypti* positive for DENV-1. Considering the *w*Mel *Ae. aegypti* mosquitoes from the two PI areas, 40–45% were infected with DENV-1, contrasting with the 10%-infected mosquitoes of the FI area. Interestingly, DENV-1 load in *w*Mel and *w*Mel-free *Ae. aegypti* was similar in mosquito bodies, except for the comparison between NR1 and PI1 (W = 198; *p* = 0.04; Figure 3A).

Regarding the exposure to the high viral titer through oral infections, all 60 mosquitoes from PI1 and PI2 were positive for DENV-1, of which 7 were *w*Mel *Ae. aegypti* and 23 were *w*Mel-free mosquitoes, and 26 were *w*Mel *Ae. aegypti* and 4 were *w*Mel-free mosquitoes, respectively. A similar outcome was observed for mosquitoes from FI, of which 20/30 were *w*Mel *Ae. aegypti* were infected with DENV-1 (96.7%). As expected, 59/60 wild mosquitoes were also infected for DENV-1 (NR1 = 30/30, NR2 = 29/30). Wild mosquitoes showed similar DENV-1 load (W = 473; *p* > 0.05; Figure 3B; Table S1). The number of DENV-1 copies in whole mosquitoes from FI was barely lower when compared with the wild mosquitoes from NR2 (W = 273; *p* = 0.02; Figure 3B, Table S1). Mosquitoes from PI1 and PI2 showed significantly different DENV-1 loads when compared to each other (W = 253; *p* < 0.05; Figure 3B) and to the controls (NR1 and NR2; W = 680; *p* < 0.05; W = 233; *p* < 0.05; W = 789; *p* < 0.05 e W = 123; *p* < 0.05; Figure 3B). Therefore, *w*Mel *Ae. aegypti* showed a similar infection rate (GLM: *Wolbachia* (Yes) estimate = −0.32, *p* = 0.818) but lower DENV-1 viral load when compared to wild mosquitoes ((GLM: *Wolbachia* (Yes) estimate = −1.3, *p*-value < 0.001; Figure 3B, Table S2). We were unable to compare the DENV-1 load of *w*Mel and *w*Mel-free mosquitoes collected in sympatry due to the small sample size (PI1 = 23 *w*Mel-free and 7 *w*Mel mosquitoes; PI2 = 4 *w*Mel-free and 26 *w*Mel mosquitoes).

### 3.4. Saliva Infectivity for DENV-1

The saliva collected from mosquitoes with DENV-1 infectivity confirmed through RT-qPCR was injected into susceptible mosquitoes from NR1, and their whole bodies were analyzed seven days after injections. The DENV-1 infection rate of the injected saliva from the studied areas is shown in Table 1. 

**Figure 4:**
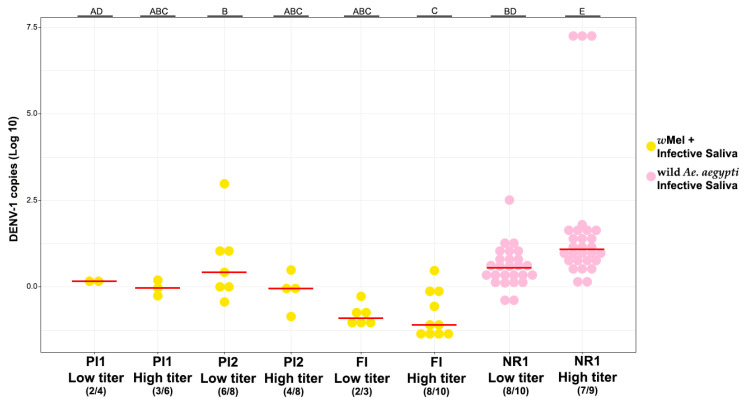
DENV-1 load in susceptible wild *Ae. aegypti* mosquitoes seven days after injecting the saliva of mosquitoes with confirmed infection of DENV-1 in the whole body. Each saliva was intrathoracically injected into 10 susceptible mosquitoes. The injected saliva was obtained from mosquitoes with and without *w*Mel and subjected to DENV-1 oral infections with low (3 × 10^4^ FFU/mL) and high titer (6 × 10^8^ FFU/mL). The number of DENV-1 infective saliva out of the total number of mosquitoes tested is in parenthesis (below the neighborhood name). Horizontal red bars represent the medians. Different letters (above the graph) indicate statistically significant differences (Wilcoxon–Mann–Whitney test; *p* < 0.05). All mosquitoes that received saliva from DENV-1-negative mosquitoes were negative.

Surprisingly, when infected with DENV-1 low titers, the viral load in wild *Ae. aegypti* who received infective saliva of *w*Mel *Ae. aegypti* was similar to the viral load observed in the control group (W = 86.5, *p* > 0.05; W = 48, *p* > 0.05; W = 52, *p* = 0.02; Figure 4, Table S1). On the other hand, when infected with the higher DENV-1 titer, there was a decrease in the viral load in *w*Mel *Ae. aegypti* saliva when compared with wild *Ae. aegypti* mosquitoes (W = 108, *p* < 0.05; W = 83, *p* < 0.05; W = 220.5, *p* < 0.0001; Figure 4, Table S1). The linear regression analyses did not detect a statistically significant correlation between DENV-1 copies in saliva-microinjected mosquitoes and the relative *w*Mel density of the mosquitoes (*F* = 2.605, *p* > 0.05, R^2^ = 0.063; Figures S2B and S3).

### 3.5. Interactions of wMel in DENV-1 Exposed Ae. aegypti

Generalized linear models revealed that when infected with low DENV-1 titer, *w*Mel *Ae. aegypti* presented a lower infection rate in their whole bodies when compared to controls (wild *Ae. aegypti*; GLM: *Wolbachia* (Yes) estimate = −1.2, *p* = 0.0005; Figure S4, Table S2). Wild and *w*Mel mosquitoes, however, had similar viral loads in their bodies (GLM: *Wolbachia* (Yes) estimate = 0.02, *p* = 0.885; Figure S4). When we analyzed the microinjected mosquitoes, both the rate of infection and the viral loads obtained seven days post injection of *w*Mel mosquito saliva were similar to the controls (saliva of wild mosquitoes; GLM: *Wolbachia* (Yes) estimates = 0.6 and −0.4, *p* > 0.1, respectively; Table S2). 

**Figure 5:**
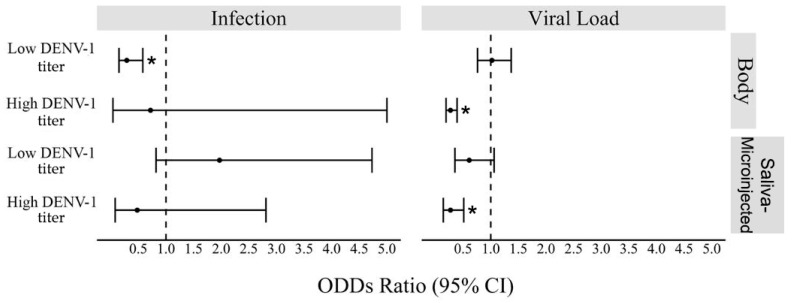
Forest plot showing the odds ratio (OR) and 95% confidence intervals for interactions of *w*Mel in DENV-1-exposed *Ae. aegypti* mosquitoes. “Infection” results are based on the number of infected and non-infected mosquitoes, and “Viral load” results are the absolute quantity of DENV-1 virus particles. Mosquito bodies were analyzed 14 days post infection (“Body”), and susceptible mosquitoes were analyzed 7 days after receiving an intrathoracic injection of saliva from a mosquito exposed to DENV-1 (“Saliva-microinjected”). Mosquitoes were fed on blood infected with DENV-1 at 3 × 10^4^ FFU/mL (“Low DENV-1 titer”) or 6 × 10^8^ FFU/mL (“High DENV-1 titer”). The asterisks denote statistically significant negative association (i.e., OR < 1.0) between having *w*Mel and body infection or viral loads.

When infected with high DENV-1 titer, *w*Mel *Ae. aegypti* mosquitoes showed similar infection rates as wild *Ae. aegypti* (GLM: *Wolbachia* (Yes) estimate = −0.32, *p*-value = 0.818; Figure S4; Table S2), but lower viral loads were observed in their whole bodies (GLM: *Wolbachia* (Yes) estimate = −1.3, *p*-value < 0.001; Figure S4) and in saliva-microinjected mosquitoes (GLM: *Wolbachia* (Yes) estimate = −1.3, *p*-value = 0.001; Figure 5, Table S2).

## 4. Discussion

In this study, the vector competence of *Ae. aegypti* was inferred in laboratory conditions by comparing the susceptibility and saliva infectivity of *w*Mel and *w*Mel-free mosquitoes collected in five localities of Rio de Janeiro, the first Brazilian city to receive *Wolbachia* deployment. We also tested if the relative density of *w*Mel in mosquito bodies influences DENV-1 infection. We subjected *w*Mel and *w*Mel-free *Ae. aegypti* to oral infections containing two DENV-1 titers (6 × 10^8^ FFU/mL or 3 × 10^4^ FFU/mL) and microinjected mosquito saliva into *w*Mel-free mosquitoes. Our results highlighted that when mosquitoes were infected with the higher DENV-1 titer, *w*Mel did not avoid viral infection in mosquitoes’ bodies and saliva, but *w*Mel mosquitoes produced lower DENV-1 loads than *w*Mel-free mosquitoes (Figure 5). On the other hand, *w*Mel mosquitoes subjected to a lower DENV-1 titer were less susceptible than *w*Mel-free mosquitoes, but once infected, *w*Mel and *w*Mel-free mosquitoes exhibited similar viral loads in the body and to saliva-microinjected mosquitoes. 

Several studies have been investigating the susceptibility of *w*Mel and *w*Mel-free *Ae. aegypti* to DENV [10,23,48,49,50,51], but there are few regarding the DENV-1 serotype [19,20,21]. In this study, we observed the superior DENV-1 infection rate (~69%) in *w*Mel mosquitoes when infected with a higher viral titer, contrasting with those infected with a lower viral titer (~22%). Taking into consideration the saliva’s infection rate, we observed that ~80% of wild *Ae. aegypti* were infective, whereas ~60% of *w*Mel *Ae. aegypti* were positive for DENV-1. Ferguson and colleagues [19] observed similar results for *w*Mel and *w*Mel-free *Ae. aegypti* from Cairns, Australia, exposed to viremic blood from acute dengue cases in Vietnam. The infection rate of mosquito body was 100%, irrespective of whether the mosquito carried *w*Mel or not for DENV-1 titers of ~10^8^ at 14 dpi, but *w*Mel mosquitoes had only ~40% of their saliva infected (lower than we observed in this study), whereas ~85% of infective saliva was observed for *w*Mel-free *Ae. aegypti*. The authors, however, did not observe infected bodies of *w*Mel and *w*Mel-free mosquitoes exposed to DENV-1 titer of ~10^5^ at 10 dpi. 

Another study in Vietnam involving four DENV serotypes has shown that 71.5% of *w*Mel-free *Ae. aegypti* and 58.7% of *w*Mel *Ae. aegypti* had their abdomens infected with DENV. Furthermore, the same held true for infectious viruses in the saliva, in which 38.5% and 22.8% for *w*Mel-free and *w*Mel *Ae. aegypti*, respectively, were infected with DENV [20]. In this study, the authors observed more mosquitoes with infective saliva when infected with DENV-1 than with DENV-4.

Flores and colleagues [21] also analyzed infection rates in *w*Mel-Cairns *Ae. aegypti* (genetic background from Cairns, Australia), *w*Mel-HCM *Ae. aegypti* (genetic background from Ho Chi Minh City, Vietnam) and *w*Mel-free mosquitoes, respectively. They observed similar infection rates of abdomen/head–thorax/saliva to Carrington and colleagues [20] and did not find any difference in the prevalence of DENV in mosquitoes with different genetic backgrounds. The authors also described for DENV-1 intrathoracic microinjection a high infection rate (94%) and a ~10^3^ viral load for *w*Mel *Ae. aegypti*, while a 100% infection rate and ~10^5^ TCID_50_/mL (for 1.2 × 10^5^ TCID_50_/mL titer) viral load were observed for Cairns *w*Mel-free *Ae. aegypti*. Concerning the Brazilian *Ae. aegypti* genetic background, Souto-Maior et al. [52] observed through DENV-1 microinjections of five viral titers (10^4^–10^8^ TCID_50_/mL) that *Wolbachia* confers a slight protection against the virus.

Divergent results on studies regarding the vector competence to DENV of *w*Mel *Ae. aegypti* might be related to the different virus genotypes [8,10,19,20,21,48,49,50,51]. In this context, the emergence of DENV variants able to replicate in *w*Mel *Ae. aegypti* and surpass the viral blockage are of general concern. For example, an amino acid substitution (*E203K*) in the DENV-1 envelope protein has increased in frequency in virus populations following 20 passages in *w*Mel *Ae. aegypti* compared to *w*Mel-free *Ae. aegypti* cell cultures. Therefore, it is highly probable that *w*Mel would exert selective pressure on dengue populations. It is still unknown, however, if these variants could efficiently replicate in the field where *w*Mel mosquitoes have been released. Future studies regarding the genome sequence of DENV variants in the field may answer this question [53].

*Wolbachia* density has been positively linked to the strength of viral blocking to RNA viruses [9,54,55,56]. For instance, in *Drosophila simulans*, it was already observed that *Wolbachia* density is important for antiviral protection [57]. Studies involving cell lines have shown almost complete DENV inhibition only if the cells have high *Wolbachia* density [55,56]. *Ae. albopictus* is naturally infected with another *Wolbachia* strain, *w*AlbB, but is unable to block DENV, which has been linked to the lower bacterium density in this species. When *w*AlbB is transinfected into *Ae. aegypti*, it is present in higher densities in mosquito tissues, and a DENV-blocking phenotype is observed [56]. In our study, *w*Mel *Ae. aegypti* from the field exhibit a lower density of the bacterium than the F1 lab-reared mosquitoes, and it may be explained by several factors such as temperature [58,59], fitness cost [13,60,61,62], and nutrition [63]. High temperatures can impact CI and *Wolbachia* density, leading to a reduction in *w*Mel frequencies and densities in *Ae. aegypti* [58,59]. Although the fitness cost of having *w*Mel is equal to both field and laboratory-reared mosquitoes [64,65], the latter are reared in optimal conditions of temperature, humidity, and nutrition, while field *Ae. aegypti* mosquitoes are subjected to adversities that may contribute to the lower *w*Mel density. Regarding the lab-reared *w*Mel *Ae. aegypti* exposed to DENV-1, there was no correlation between relative *w*Mel density and virus infection of the mosquito’s body or saliva (Figure 5), as already reported [66,67]. Even though *w*Mel density does not appear to influence DENV-1 infection, tissue-specific (ovaries, salivary glands, and saliva’s infectivity) analysis could shed a brighter light on this issue.

We observed contrasting scenarios in Rio regarding the *w*Mel geographical invasion and relative density in mosquitoes and the viral blocking phenotype of *Ae. aegypti*. FI is an isolated neighborhood with low human density where *w*Mel deployment was completed in 2015 and had the highest *w*Mel frequency and density. Moreover, its *w*Mel mosquitoes had the lowest DENV-1 loads (Figure 1, Figure 2 and Figure 5). PI1, a highly urbanized and human-populated area surrounded by places with few or no *Wolbachia* deployments, where *Wolbachia* deployments occurred in early 2019, *w*Mel is still in low prevalence, and mosquitoes had high viral loads in their bodies and saliva. In PI2, where *w*Mel releases occurred in late 2019 and the bacterium is highly prevalent, the mosquitoes also had high viral loads in the saliva. Therefore, even though there is not a positive linear relationship between *Wolbachia* density and DENV-1 infection, the results observed in FI suggest there may exist a *Wolbachia* density threshold, which allows better protection for the arboviruses. Another hypothesis would be that the positive relationship between *Wolbachia* density and protection may occur in some strategic tissues for *Wolbachia* but not in others, and this outcome is masked in this study once we analyze the whole mosquito bodies. 

The release of *Ae. aegypti* with *Wolbachia* has been a promising strategy to mitigate the transmission of arboviruses such as DENV, ZIKV, and CHIKV in urban settings. Nonetheless, the success of *Wolbachia* as a strategy to reduce arboviruses varies given the complexity of each city’s environment and factors, such as the insecticide resistance of *Ae. aegypti* carrying *Wolbachia*, native field vector population sizes, and the maintenance of MT and CI in field conditions [30,36,68,69,70,71]. It is relevant to bear in mind that *w*Mel deployment has a significant public health impact and likely achieves disease elimination in low/moderate arboviral transmission scenarios, but it seems to be less efficient to control the transmission in highly epidemic regions, particularly considering DENV-1 [19]. Studies in Rio de Janeiro, Indonesia, and Australia have shown a reduction in dengue cases using the *w*Mel strain. Australia had a great outcome with a reduction of >95% of DENV cases. In Indonesia, a better outcome was observed when compared to Rio’s study, showing a reduction of 77% of the DENV cases against 38% [26,30]. These differences may be associated not just with the moderate *w*Mel introgression into wild *Ae. aegypti* population (average 40%) in Rio de Janeiro after 4–5 years of *Wolbachia* deployments across the city [30,42], but also to the potential DENV leakage in the saliva of *Wolbachia*-infected individuals that could reduce the epidemiological effectiveness of this strategy. It is also important to highlight that there are differences in the incidence rate (IR) of dengue cases in these countries. In 2022, Australia had an IR of 1.55 cases of dengue (per 100,000 habitants), while Indonesia showed an IR of 45.7 cases, and Brazil had an impressive IR of 1097 cases [72,73]. Therefore, these distinct epidemiological scenarios may explain the different success rates in the reduction of dengue cases in these countries. Continuous monitoring of *Wolbachia* in the field and dengue cases in humans remains of utmost importance to identify any changes in the epidemiological scenario so action plans can be implemented in time.

## 5. Conclusions

This study provides insights into the tripartite interaction involving the *w*Mel strain of *Wolbachia*, *Ae. aegypti* mosquitoes and DENV-1 in the highly epidemic scenario of Rio de Janeiro, Brazil. The presence of *w*Mel did not avoid viral infection in mosquitoes’ bodies and saliva when subjected to a higher DENV-1 titer, but *w*Mel mosquitoes exhibited lower viral loads than wild mosquitoes. Regarding the lower DENV-1 titer, *w*Mel mosquitoes were less susceptible than *w*Mel-free mosquitoes, but once infected, *w*Mel and wild mosquitoes exhibited similar viral loads in the body and the saliva-microinjected saliva. Furthermore, DENV-1 infection and viral titer in the body or in saliva-microinjected mosquitoes seem to not be related to *w*Mel density. Future studies concerning the vector competence of *w*Mel and wild *Ae. aegypti* with varied genetic backgrounds are necessary to check the impact on *w*Mel’s strategy to replace wild *Ae. aegypti* populations and diminish arbovirus transmission in the long term.

## Figures and Tables

**Table 1:** DENV-1 infection rate per area observed in wild *Ae. aegypti* mosquitoes 7 days post saliva microinjection with low titer (3 × 10^4^ FFU/mL) and high titer (6 × 10^8^ FFU/mL). The numbers inside parentheses indicate the number of DENV-1-positive mosquitoes out of the total mosquitoes tested per area.

	DENV-1 Infection Rate	
Areas	Low Titer	High Titer
**NR1**	80% (8/10)	77.77% (7/9)
**PI1**	50% (2/4)	50% (3/6)
**PI2**	75% (6/8)	50% (4/8)
**FI**	66.66% (2/3)	80% (8/10)

## Data Availability

Data are contained within the article and supplementary materials.

## Supplementary Materials

The following supporting information can be downloaded at: https://www.mdpi.com/article/10.3390/v16020214/s1, Figure S1: Relative density of *w*Mel *Ae. aegypti* mosquitoes separated into “FIELD” and “LAB” groups (A) and in field localities and laboratory F1 generation (B). Horizontal red bars represent the medians. Green, yellow, and pink circles represent samples infected with *w*Mel from FI, PI1, and PI2, respectively. Figure S2: Regression analyses comparing relative *w*Mel density in whole *Ae. aegypti* mosquitoes with (A) DENV-1 load in whole mosquitoes at 14 dpi, and (B) DENV-1 load in susceptible mosquitoes seven days post intrathoracic injection of mosquito saliva. Both regression analyses were not statistically significant (*F =* 2.354, *p* > 0.1289 and *F =* 2.605, *p* > 0.1202, respectively). Figure S3: Relative density of *w*Mel in *Ae. aegypti* mosquitoes infected with DENV-1 at low (3 × 10^4^ FFU/mL) and high titer (6 × 10^8^ FFU/mL). The numbers inside parentheses (below the neighborhood name) indicate the number of *w*Mel-positive mosquitoes out of the total mosquitoes tested. Different letters (above the graph) indicate statistically significant differences in *w*Mel density (Wilcoxon–Mann–Whitney test; *p* < 0.05). Horizontal red bars represent the medians. Purple circles represent samples infected with *w*Mel and untested for infective DENV-1 in the saliva. Yellow and green circles represent the samples with and without infective DENV-1 particles in the saliva, respectively. Figure S4: Number of DENV-1 copies in the whole body observed in *w*Mel, *w*Mel-free, and wild *Ae. aegypti* mosquitoes 14 days post infection with low titer (3 × 10^4^ FFU/mL) (A) and high titer (6 × 10^8^ FFU/mL) (B). The numbers inside parentheses (below the neighborhood name) indicate the number of DENV-1-positive mosquitoes out of the total mosquitoes tested. Different letters (above the graph) indicate statistically significant differences (Wilcoxon–Mann–Whitney test; *p* < 0.05). Horizontal red bars represent the medians. Purple and gray circles represent mosquitoes infected with *w*Mel and wild, respectively, but untested for infective DENV-1 in the saliva. Yellow and pink circles represent mosquitoes with and without *w*Mel, respectively, with infective DENV-1 in the saliva. Mosquitoes with and without *w*Mel with DENV-1 non-infective saliva are represented by green and blue circles, respectively. All negative controls for DENV-1 infection were negative. Table S1: Results of the Wilcoxon–Mann–Whitney pairwise comparisons related to DENV-1 infection in the body and saliva and density of *Wolbachia* of *Ae. aegypti* mosquitoes. Table S2: Results of the generalized linear models (GLMs) related to DENV-1 infection in the body and saliva and density of *Wolbachia* of *Ae. aegypti* mosquitoes.
