# Supplementary figures and images for: DENV-1 Titer Impacts Viral Blocking in wMel Aedes aegypti with Brazilian Genetic Background
Source: Viruses. 2024 Jan 31;16(2):214. doi: 10.3390/v16020214 (PMC10891765; doi:10.3390/v16020214)

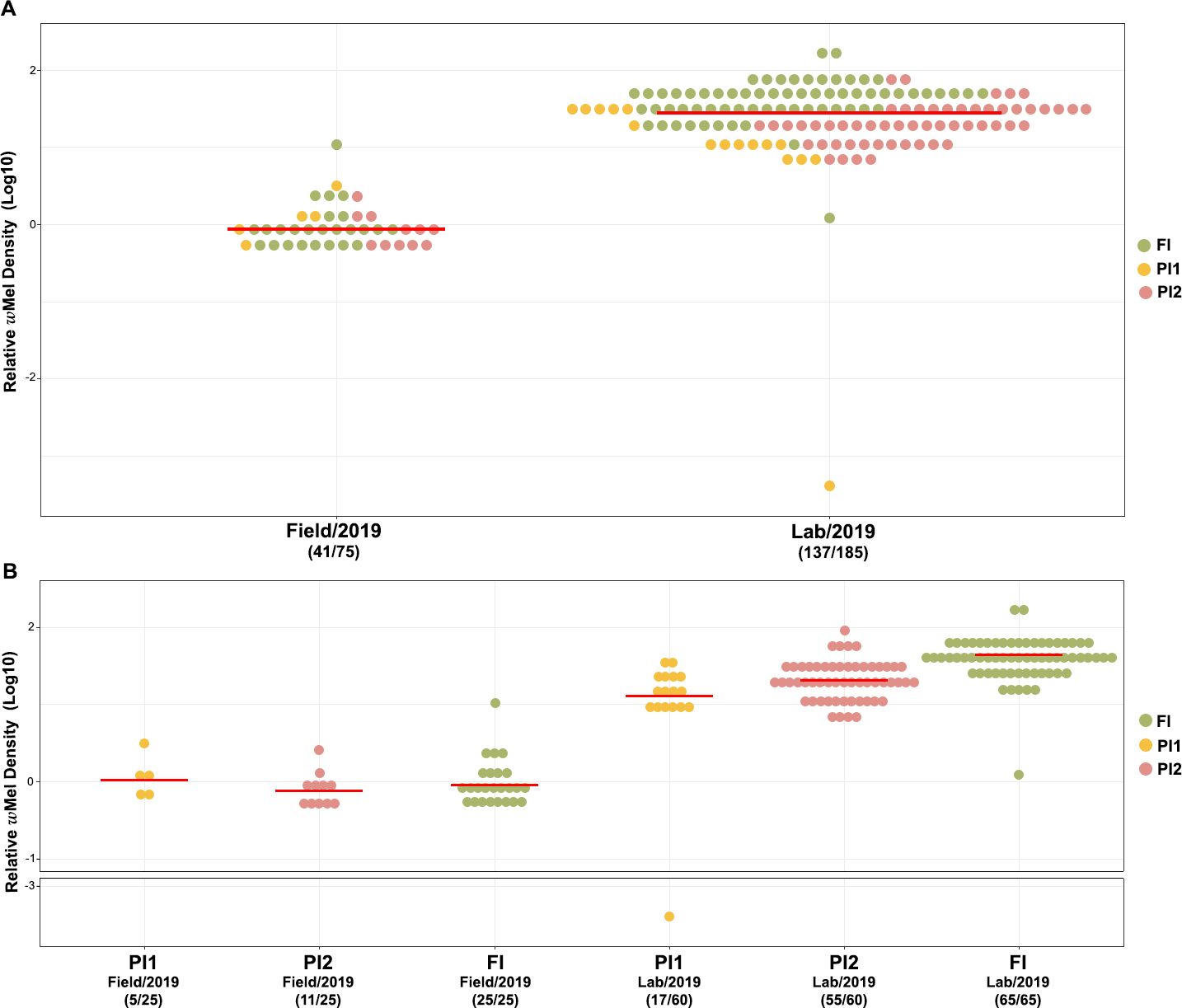

Supplement: Supplementary file 1 [file viruses-16-00214-s001.zip › Figure_S1.tif]

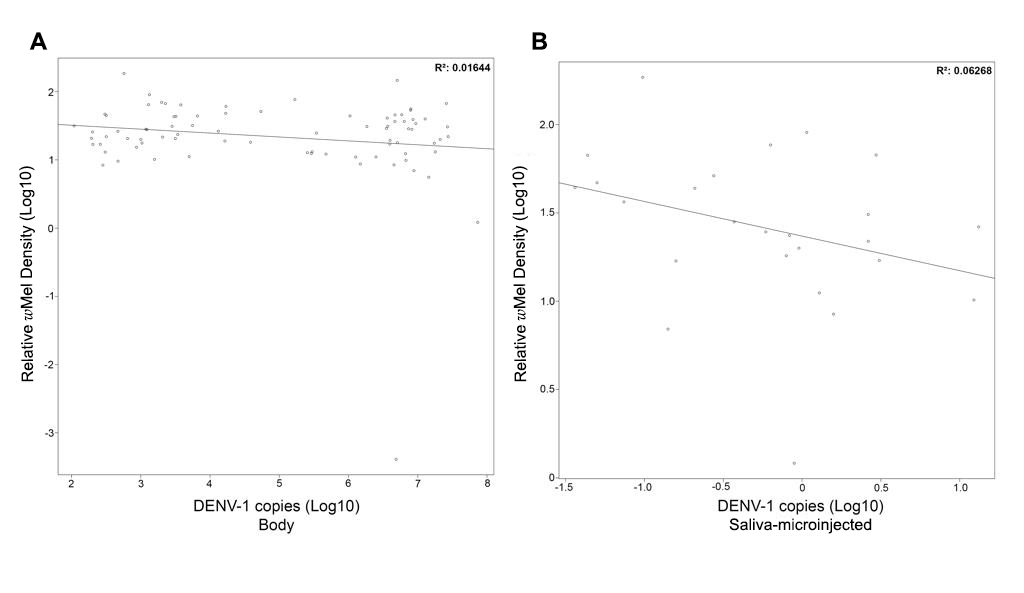

Supplement: Supplementary file 1 [file viruses-16-00214-s001.zip › Figure_S2.tif]

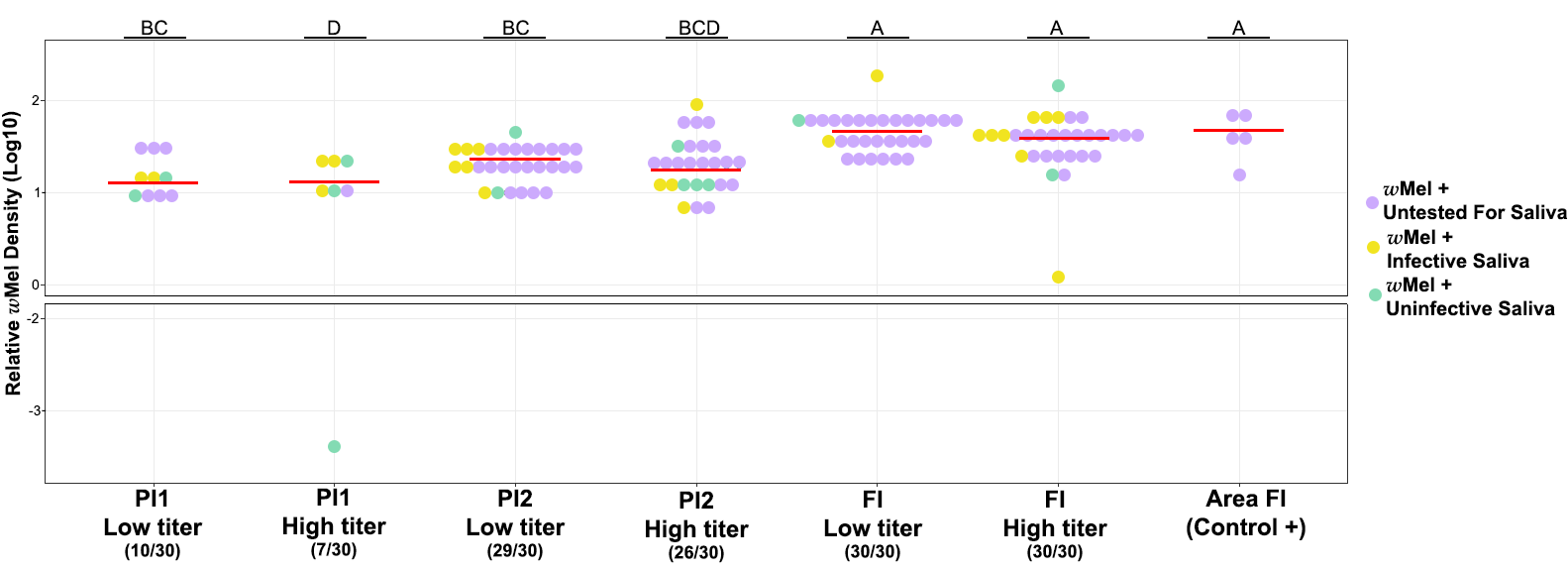

Supplement: Supplementary file 1 [file viruses-16-00214-s001.zip › Figure_S3.tif]

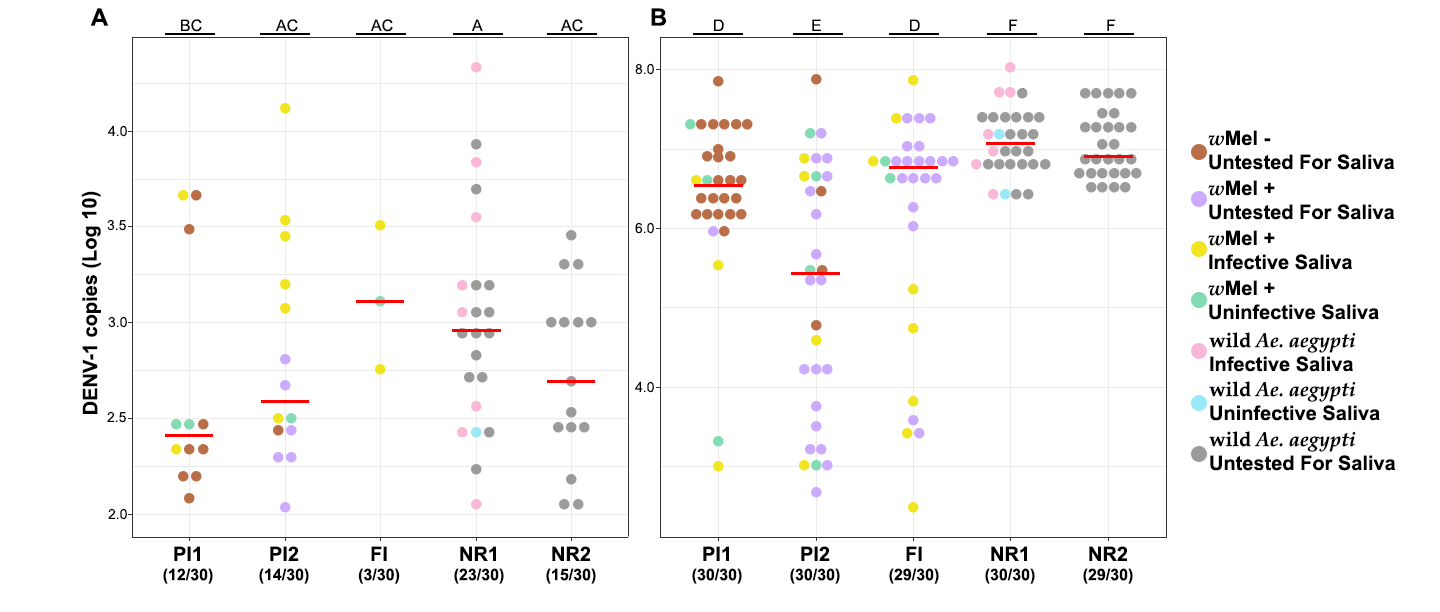

Supplement: Supplementary file 1 [file viruses-16-00214-s001.zip › Figure_S4.tif]
